# Link budget analysis of bi-directional LEO and GEO optical feeder links advancing the beam wander model’s accuracy

**DOI:** 10.1038/s41598-024-59198-x

**Published:** 2024-04-13

**Authors:** Carla Cantore, Davide Monopoli, Angelo Altamura, Alberto Mengali, Marco Grande, Antonella D’Orazio

**Affiliations:** 1 Department of Electrical and Information Engineering, Polytechnic University of Bari, 70126 Bari, Italy; 2 European Space Agency, ESTEC, 2201 AZ Noordwijk, The Netherlands

**Keywords:** Electrical and electronic engineering, Optical physics

## Abstract

The telecommunications of the future rely on the concept of a three-dimensional architecture able to integrate terrestrial and non-terrestrial networks with the goal to ensure a reliable and high-speed connectivity to users located anywhere. In this context, free space optical communications constitute a candidate technology for feeder links, thanks to their advantages in terms of bandwidth and achievable data rates. Nonetheless, due to the propagation impediments encountered by an optical beam travelling through atmosphere, flexible and accurate instruments able to support the design of optical feeder links are needed. Therefore, in this paper a link budget numerical tool able to meet these requirements is presented and the link budget analysis for real optical feeder links is performed demonstrating its prediction accuracy by means of the comparison with experimental results for both low Earth orbit and geostationary Earth orbit based configurations. Finally, the limits of the conventional beam wander model are analyzed and overcome.

## Introduction

Free space optical (FSO) communications, consisting in the line-of-sight transmission of a modulated optical beam between a transmitter and a receiver, are gaining more and more attention as a possible solution to cope with the ever-increasing demand for higher bandwidths and capacities arising from the spread of multimedia services as well as from the definition of new use cases for future fifth generation and Beyond (B5G) and sixth generation (6G) networks^1^. Besides the communication performance improvement that comes with every new generation of mobile networks, one of the B5G/6G main goals is to overcome the current lack of a reliable and broadband connectivity serving users living outside urban areas, but also to improve the resilience of the existing network infrastructures in response to natural disasters and emergency situations^2^. Therefore, future mobile networks will rely on the new concept of 3-D networks made up of terrestrial and non-terrestrial nodes, such as satellites, unmanned aerial vehicles (UAVs), and high-altitude platforms (HAPs), allowing to reach a high-capacity worldwide connectivity.

In the context of non-terrestrial networks (NTNs), the possibility to employ the FSO technology is under investigation due to its numerous advantages, among which it is necessary to mention the availability of a huge amount of spectral bandwidth, in the order of Terahertz, not subjected to regulations. The latter can allow to reach the high data rates required by the actual and, above all, future expected traffic demand that conventional radiofrequency (RF) links are not able to meet, but also to overcome the congestion experienced by the RF licensed spectrum. Moreover, optical wireless communications (OWCs) offer less power consumption for the same throughput and smaller system sizes compared to the RF counterpart^3^, other than a lower beam divergence that, considering only geometric effects, leads to a higher signal intensity at the receiver and makes it more difficult for a malicious user to perform an attack.

In addition, the European Space Agency (ESA), within its ongoing High Throughput Optical Network (HydRON) project^4^, has already identified FSO technology as the solution to develop an all-optical global network through the seamless integration of the existing terrestrial optical transport networks (OTNs) with a new space-based optical network architecture able to guarantee the same performances, in terms of capacity and throughput, delivered by the fiber-based OTN. This new paradigm implies the adoption of bi-directional FSO links between optical ground stations (OGSs) and satellites, i.e., optical feeder links (OFLs), as well as optical links among satellites, i.e., optical inter-satellite links (OISLs)^5^. The latter are characterized by a more mature technology already employed, for example, in the European Data Relay System (EDRS), and deployed in the SpaceX’s Starlink constellation^6^. The former are more challenging to implement so that several demonstrations were carried out, but no operational OFL is available yet^7^. This is due to the propagation channel, i.e., while an OISL runs far from the terrestrial atmosphere, an OFL involves the propagation of the optical beam both through free space and atmosphere, being the latter the limiting factor in the achievable system performances due to several phenomena affecting the optical beam differently from the ones experienced by a RF signal. Among them, the most detrimental one is represented by atmospheric turbulence which induces several effects on the optical beam, such as scintillation and beam wander. Therefore, in order to support future systems design activities, it is necessary to estimate the achievable performances through the accurate evaluation of typical figures of merit.

In this paper, a flexible numerical tool able to support the design of both ground-to-space and space-to-ground OFLs is proposed. In particular, the tool focuses on the link budget estimation which allows to evaluate the feasibility of a link from the computation of the received optical power taking into account the influence of all the system components, i.e., the transmitter, the channel, and the receiver, on the optical beam propagation through gain and loss terms.

In literature, several examples of link budget analysis for OFLs have been reported^8–14^ that show several limits.

The majority of the existing literature works is focused on the link budget calculation for specific scenarios of interest, often modeling only one propagation direction, i.e., only uplink^12^ or only downlink^13^, while others consider just one propagation theory^10,12,14^. The numerical tool proposed in this paper presents several advantages and novelties with respect to them. First of all, the accurate modeling of turbulence-induced phenomena has been fulfilled, considering the differences between uplink and downlink propagation directions, as well as two different propagation theories, i.e., the Rytov theory, valid only under weak turbulence, and the extended Rytov theory, valid under all turbulence regimes.

Moreover, some turbulence-related quantities are often neglected or evaluated only through analytically simpler but not so accurate models, although they play an important role when it comes to evaluate the performances of FSO links through atmosphere.

Among them, beam wander, which leads to pointing errors and increased scintillation, plays a major role in the achievable system performances, as will be deeply explained in the next sections. The proposed model evaluates the pointing loss deriving from beam wander as well as the induced scintillation aggravation, which are overlooked in^8–10,14^. Furthermore, the tool allows to overcome the limitation related to the adoption of a coarse link distance approximation in the conventional model describing the turbulence-related beam wander effect, through the possibility of substituting it with: (1) a more accurate geometrical approximation; or (2) with the exact link distance value. The integration of this tool capability stems from a novel investigation about the impact that the link distance accuracy has on the beam wander-related quantities. To the best of author’s knowledge, this is the first time that this analysis has been addressed.

High pointing accuracies are crucial to establish OFLs. Indeed, due to the narrow optical beam divergence and to the Gaussian laser irradiance profile, transmitter and receiver alignment must be ensured. Our link budget model differentiates itself from existing ones by the capability to evaluate the losses deriving from both deterministic pointing errors, e.g., misalignments in the transmitter optics, and random errors, such as beam wander and mechanical vibrations. Indeed, most literature works neglect pointing errors^8,9,14^, or evaluate their impact in a restricted way. For example^10^, only models deterministic pointing errors, unlike^13^ that only considers random pointing jitter, while^11,12^ apply an approximated model which does not distinguish between random and deterministic errors.

Another critical aspect to evaluate the OFL availability is represented by perturbed sky effects. Despite this^10,12,13^, do not include the losses deriving from hydrometeors in their link budgets. Several empirical models can be found in literature regarding fog, clouds, rain, and snow losses, whose applicability depends on the considered scenario. Thus, the proposed numerical tool gives the flexibility to choose among different models to compute the losses related to perturbed sky effects, differently from other works, such as^11^, that do not offer this kind of versatility.

In a previous paper, we developed a preliminary optical link budget model^15^. In this paper, we present a novel link budget numerical tool for OFLs, which accounts for the accurate modeling of beam wander as well as deterministic and random pointing errors, giving the possibility to analyze the transmission of both tracked and untracked beams. Moreover, the numerical tool is able to evaluate link budgets of dynamic scenarios involving low Earth orbit (LEO) satellites. This is done reconstructing the satellite orbit and computing the link budget for sampled time instants, spanning the link duration. In such a way, the impact of the varying link geometry on the system performance can be established.

Thanks to these capabilities, the accuracy of the proposed link distance approximation has been demonstrated against varying elevation angles, consequently proving the relevant improvement in the beam wander and link budget estimations performed by the tool.

The numerical tool accounts for several models quantifying the turbulence strength, derived from experimental campaigns. This feature, together with the previous ones, allows to reliably estimate OFLs performances, as demonstrated by the analysis of real experimental scenarios.

In conclusion, the tool has been developed paying attention to guarantee flexibility, which has been reached giving the possibility to the user to freely define the scenario under analysis through the definition of many input parameters, as well as allowing to choose between different available mathematical models to evaluate the losses related to each propagation phenomenon.

The paper is organized as follows. “System and channel models for OFLs” section describes the electromagnetic phenomena and the implemented models involved in the optical beam propagation through atmosphere and free space together with those related to the evaluation of the losses. In addition, the contributions of the receiver and transmitter to the link budget are shown. “Link budget analysis for experimental OFLs” section provides the validation of the proposed numerical tool based on the comparison of real data derived from OFLs experimental demonstrations and measurements, highlighting the tool accuracy and sweep capabilities. “Impact of link distance on the beam wander for experimental OFLs” section investigates the impact of the aforementioned different link distance evaluations on the quantities related to beam wander. Finally, conclusions and future works are discussed in “Conclusions” section.

## System and channel models for OFLs

An OFL can be seen as a point-to-point FSO link in which the transmitter modulates the information bits onto an optical carrier that is collimated and sent, by means of a telescope, through the channel. At the receiver, a telescope collects the incident beam through its lens and focuses it onto a photodetector which converts the optical signal into an electrical one that can be elaborated and demodulated to retrieve the transmitted information data.

**Figure 1:**
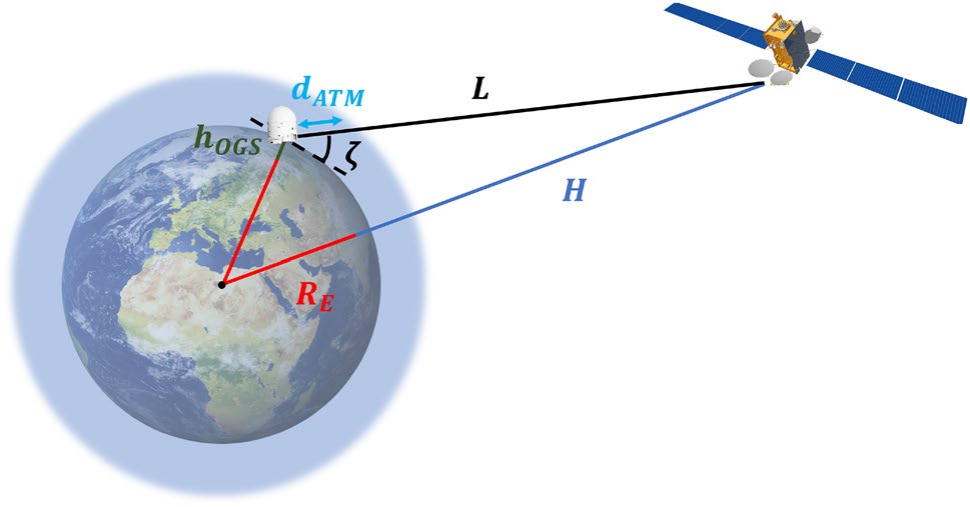
Optical feeder link geometry.

The geometry of an OFL between an OGS and a satellite is shown in Fig. 1, in which $L$ is the link distance, i.e., the distance between the OGS and the satellite, $H$ and $h_{OGS}$ are the satellite and OGS altitudes, respectively, being $\zeta$ the elevation angle between them. $R_E$ is the mean Earth radius and $d_{ATM}$ is the length of the atmosphere portion crossed by the optical beam.

This section aims to provide a comprehensive description of the way in which each element composing the overall OFL affects the beam propagation and, consequently, the received optical power. This analysis requires to distinguish between the two possible propagation directions, i.e., ground-to-space or uplink and space-to-ground or downlink. This is due to the channel asymmetry since an uplink beam travels first across the atmosphere and then through the free space, as opposed to a downlink one.

Each electromagnetic phenomenon or system component taking part in the link budget evaluation is discussed, along with the implemented mathematical models.

### Free space

The free space portion of the propagation channel induces only diffraction of the optical beam while it propagates. Therefore, an attenuation term $L_{FS}$ related to the growing beam divergence experienced by the wave must be accounted for in the link budget equation. According to the Friis law

1 $$\begin{aligned} L_{FS} = \left( \frac{\lambda }{4 \pi L}\right) ^2 \end{aligned}$$

where $\lambda$ (m) is the operating wavelength and $L$ (m) is the link distance.

### Absorption and scattering

The terrestrial atmosphere is made up of gases and particles whose density decreases with altitude. Their interaction with the optical beam can lead to absorption and scattering of some photons resulting in loss and angular redistribution of the beam energy, respectively. Both phenomena strictly depend on wavelength and on the concentration and size of the atmosphere molecules for a specific location.

Absorption and scattering are commonly grouped together under the name of extinction, representing the attenuation experienced by a wave propagating through the atmosphere. The related loss, $\tau _{ATM}$ (dB), is quantified by the Beer–Lambert law^16^

2 $$\begin{aligned} \tau _{ATM} = 10\log _{10} \exp \left[ - \alpha (\lambda ) d_{ATM}\right] \end{aligned}$$

in which $d_{ATM}$ (m) is the length of the atmosphere portion crossed by the optical beam and $\alpha (\lambda )$ (m$^{-1}$) is the extinction coefficient^16^.

### Optical turbulence

The heating of the Earth surface due to the incident solar radiation is the cause of random air temperature variations. The latter, combined with random pressure variations, induce optical turbulence, i.e., stochastic fluctuations of the atmosphere refractive index, both in space and time^17^. This phenomenon is described by the Kolmogorov theory, which follows a statistical approach, and leads to three main effects involved in the optical beam propagation, i.e., scintillation, beam wander, and additional beam spreading. To understand how these effects are differently involved in the two transmission directions, it is necessary to mention that optical turbulence comes with the formation of turbulent eddies whose sizes are comprised between an inner scale of turbulence $l_0$ and an outer scale $L_0$^17^.

The turbulence entity is quantified by the refractive index structure parameter $C_n^2(h)$ (m$^{-2/3}$) which, for a slant path as the one involved in an OFL, depends on the altitude $h$ (m). Even if several mathematical models exist in literature for this parameter, the most used is the Hufnagel-Valley (HV) one, expressed as^17^

3 $$\begin{aligned} C_n^2 (h) = 0.00594\left( \frac{w}{27}\right) ^2 (10^{-5} h)^{10} \exp \left[ -\frac{h}{1000}\right] + 2.7\cdot 10^{-16} \exp \left[ -\frac{h}{1500}\right] + A\exp \left[ -\frac{h}{100}\right] \end{aligned}$$

where $w$ (m/s) is the root mean square wind speed and $A$ (m$^{-2/3}$) is the nominal value of the structure parameter at ground level.

The values $A=1.17\times 10^{-14}$ m$^{-2/3}$ and $w=21$ m/s, recommended by the International Telecommunication Union (ITU) for nighttime observations^18^, lead to the so-called HV-5/7 model^19^. To represent different turbulence strengths, multiples of the HV-5/7 model, indicated as MxHV-5/7 can also be considered.

The proposed numerical tool also implements a modified version of the HV model accounting for the ground layer atmospheric effects on an OGS located at an altitude $h_{OGS}$ (m) above the mean sea level^12^.

Another turbulence-related parameter is the atmospheric coherence length $r_0$ (m), also-called Fried’s parameter, whose expression depends on the wave model. Even if the transverse mode emitted from a laser is typically Gaussian, the downlink beam is well approximated by a plane wave^17^, while the uplink one by a spherical wave^20^. In the first case, the Fried’s parameter can be evaluated as^3^

4 $$\begin{aligned} r_0=\left[ 0.423 k^2 \sec \,(\xi ) \int _{h_{OGS}}^H C_n^2 (h) \,dh \right] ^{-\frac{3}{5}} \end{aligned}$$

where $\xi$ ($^\circ$) is the zenith angle between the OGS and the satellite located at an altitude $H$ (m) and $k=2\pi /\lambda$ is the wavenumber. Instead, for a spherical wave^3^

5 $$\begin{aligned} r_0=\biggl [0.423 k^2 \sec \,(\xi ) \int _{h_{OGS}}^H C_n^2 (h) \cdot \left( 1-\frac{h-h_{OGS}}{H-h_{OGS}}\right) ^{\frac{5}{3}} \,dh \biggr ]^{-\frac{3}{5}}. \end{aligned}$$

A smaller value of $r_0$ is associated with a stronger turbulence level.

### Scintillation

When the eddies size is comparable with the beam size, redistribution of the energy within the beam occurs resulting in irradiance fluctuations, known as scintillation.

While scintillation does not change the average received optical power, it induces temporal variations in its instantaneous value that can still degrade the OFL performance. Indeed, it can eventually lead to fading of the received signal below the receiver sensitivity, reducing the link availability^17^. The latter is related to the fraction of operating time in which link failure occurs and, depending on the application, a specific link availability is required.

Scintillation is typically quantified through the scintillation index, i.e., the normalized variance of irradiance, whose mathematical expression changes based on the propagation direction for a slant path.

The scintillation index, in the most general case, is the sum of a radial term and a longitudinal one. Nonetheless, considering that in uplink the transverse correlation width of irradiance $\rho _c$ (m) will be certainly larger than the receiver aperture diameter $D_R$ (m)^17^, it is possible to assume the receiver as a point one and, consequently, to consider only the longitudinal component of $\sigma _I^2$. On the contrary, in the downlink case $\rho _c$ can be smaller than $D_R$ implying that the link will benefit from aperture averaging at the receiver, reducing the scintillation impact.

The expression of the scintillation index depends also on the turbulence regime. For weak turbulence regime, the Rytov theory regarding the beam propagation through turbulence can be adopted which is also more computationally manageable. Instead, outside this regime, the expression derived from the extended Rytov theory must be considered, given that the latter is valid in all turbulence regimes. The expression of the transverse correlation width also varies according to the two theories hence, given the chosen model and propagation direction as input parameters, the proposed numerical tool evaluates $\sigma _I^2$ through the proper expression, automatically verifying for the downlink case if $\rho _c$ is smaller than $D_R$ and, if so, applying the averaged scintillation index expression. All the implemented expressions for the scintillation index can be found in^17^.

The evaluation of the scintillation index is essential to compute the signal-to-noise ratio (SNR) and bit error rate (BER) in a FSO link, as stated in^21^.

To counteract turbulence-induced fading, a proper link margin, i.e., a surplus of optical power, should be considered at the transmitter side. The choice of an adequate link margin can be done evaluating the probability that the received power falls below a given threshold^22^. Thus, a mathematical model for the probability density function (PDF) of the received irradiance is required.

An expression for the scintillation-related fade margin $L_{SI}$ (dB) has been derived in^23^, according to which

6 $$\begin{aligned} L_{SI}=4.343\biggl \{{{\,\textrm{erf}\,}}^{-1}\,(2\rho _{thr}-1)[2\ln \,(\sigma _I^2+1)]^{\frac{1}{2}} - \frac{1}{2}\ln \,(\sigma _I^2+1)\biggr \} \end{aligned}$$

where $\rho _{thr}$ is the desired fraction of outage time, i.e., the probability that the received power falls below the considered threshold, and $\sigma _I^2$ is the scintillation index. This model assumes a lognormal PDF for the received power after aperture averaging. Moreover, some evidence that the lognormal distribution for power holds even when the received intensity PDF does not follow a lognormal behavior, as in presence of strong turbulence, exists^14,23^.

The model of Eq. (6) does not consider that deep fades can cause loss of clock, requiring a certain time period to achieve resynchronization of the bit stream, during which data are lost. Thus, an increase in the mean BER will be experienced. Moreover, it does not account for the additional fading coming from pointing and tracking errors.

In conclusion, the presented model has been implemented in the tool, together with an approximated version valid only in specific ranges of $\rho _{thr}$ and $\sigma _I^2$^23^, to give an estimation of the required fade margin. Nonetheless, the computed value must not be considered sufficient to counteract turbulence and achieve the desired link availability, especially in scenarios where the lognormal model for the received power does not hold. Other mitigation techniques, such as forward error correction codes and interleaving, must be employed to achieve the desired BER^21^.

### Beam wander

Eddies bigger than the beam size induce random displacements of the beam hot spot at the receiver, known as beam wander. This effect can be neglected in downlink since, due to the cited asymmetry, the downlink beam will spread in free space reaching the atmosphere with a diameter larger than the typical outer scale of turbulence. Instead, in uplink, the beam will stay smaller than $L_0$ through the atmosphere path suffering from beam wander, with possible displacement values up to several hundred meters^17^.

In detail, eddies bigger than the atmospheric coherence length are related to phase fluctuations. Instead, eddies bigger than beam size but smaller than $r_0$ induce further irradiance fluctuations, consequently leading to an increased on-axis scintillation index.

Applying the reciprocity principle, beam wander at the receiver plane can be modeled as if it arises from a random tilt at the transmitter plane.

The average displacement of the received beam from the boresight is quantified through the beam wander variance $\langle {r_c^2}\rangle$ that, for a Gaussian collimated beam and assuming an infinite outer scale of turbulence, is conventionally evaluated as^17^

7 $$\begin{aligned} \langle {r_c^2}\rangle =0.54(H-h_{OGS})^2\sec ^2\,(\xi )\left( \frac{\lambda }{2 W_0}\right) ^2\left( \frac{2 W_0}{r_0}\right) ^{\frac{5}{3}} \end{aligned}$$

where $W_0$ (m) is the transmitter beam radius that, if not known, can be evaluated by the proposed numerical tool considering its relationship with the transmitter aperture diameter $D_T$ (m), that for a Gaussian beam is^17^

8 $$\begin{aligned} D_T^2=8 W_0^2. \end{aligned}$$

Angular beam wander $\theta _{BW}$ (rad) gives the same information of beam wander variance, but seen as an angular tilt at the transmitter side, and it is expressed as^12^

9 $$\begin{aligned} \theta _{BW}=\frac{\sqrt{\langle {r_c^2}\rangle }}{L}. \end{aligned}$$

The conventional beam wander variance expression of Eq. (7) considers the following approximation for the link distance^17^:

10 $$\begin{aligned} L\cong (H-h_{OGS})\sec \,(\xi ). \end{aligned}$$

The approximation of Eq. (10), assuming a flat Earth model, is commonly adopted in turbulence-related expressions and literature link budget models. Since this approximation appears to be coarse, especially for high zenith angles, the proposed numerical tool allows also to evaluate the beam wander variance considering a more accurate approximation, derived as described in^24^, but without neglecting the OGS altitude

11 $$\begin{aligned} L\cong -(R_E+h_{OGS})\sin \,(\zeta ) +[(R_E+H)^2-(R_E+h_{OGS})^2\cos ^2\,(\zeta )]^\frac{1}{2}. \end{aligned}$$

In Eq. (11), $\zeta$ = $90-\xi$ [$^\circ$] is the elevation angle and $R_E$ (m) is the mean Earth radius. In addition, there is also the possibility in the tool to evaluate Eq. (7) considering the exact link distance value, if it is known, in place of its approximation. Considering that the beam wander variance is directly proportional to the link distance squared, the tool capability to accurately evaluate the link distance relevantly improves the beam wander estimation.

It is important to point out that Eq. (9) is valid only if the same link distance is used for both the numerator and the denominator therefore, substituting the coarse approximation for the numerator and a different value for the denominator, will lead to a wrong evaluation of the beam wander effect.

**Figure 2:**
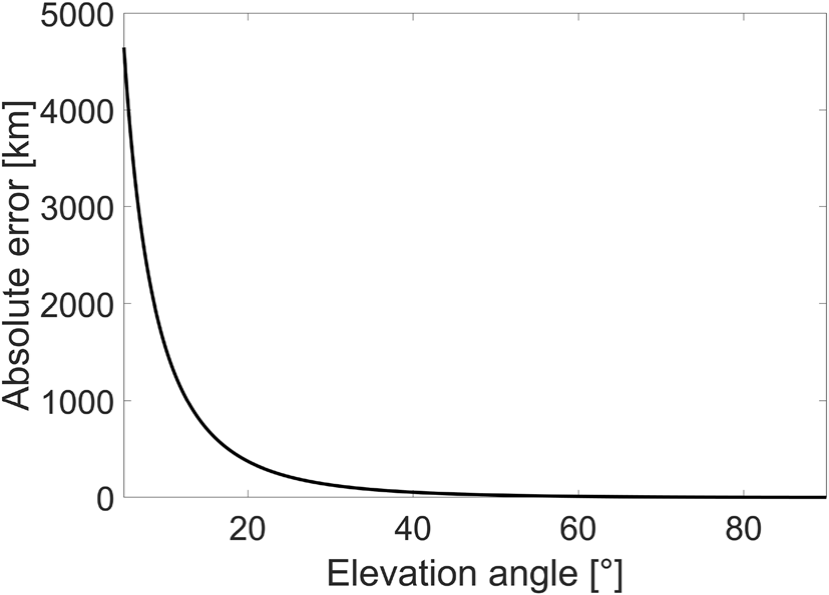
Absolute error between the link distances evaluated through the conventional and more accurate approximations for LEO scenario.

Figure 2 shows the absolute error between the two approximations given in Eqs. (10) and (11) as a function of the elevation angle. In particular, the plot considers an OGS at an altitude of 122 m, as the one located in Koganei and operated by the National Institute of Information and Communications Technology (NICT), and a LEO satellite at an altitude of 610 km. The plot highlights that the difference between the two approximations is higher for smaller elevation angle, while it decreases as the elevation angle increases. A zero error can be achieved only for an elevation angle $\zeta =90^\circ$ since, only in this case, Eqs. (10) and (11) return the same distance value. Further analysis on how the different approximations affect the beam wander evaluation will be performed in “Impact of link distance on the beam wander for experimental OFLs” section.

Beam wander leads to the worsening of time-varying power fades due to the increased scintillation. In addition, both the movement of the short-term beam centroid and the motion of the hot spot around the beam centroid can be observed at the satellite. Thus, beam-wander induced pointing errors occur. Due to these effects, beam wander must be properly characterized and considered in link budget analysis, especially if countermeasures to mitigate it are not enforced at the OGS.

The simplest way to reduce beam wander consists of increasing the beam divergence, so that the probability of missing the target decreases. However, this approach leads to a reduced received power. Therefore, the preferred mitigation technique is to use a fast-tracking transmitter to track the beam.

Tracking can be seen as a first-order adaptive optics (AO) system^25^. Indeed, the phase of an optical wave passing through a circular aperture can be represented by an infinite summation of modes described by the orthogonal basis given by the Zernike polynomials. The latter can be related to typical aberrations involved in optical systems. In particular, the first Zernike mode is associated with the averaging effect of a circular receiving aperture, while the next two modes represent the vertical and horizontal tilts of the incoming wavefront^26^. Thus, lowest-order AO, i.e., tilt compensation, allows to compensate for beam wander correcting the first three spatial modes.

In uplink OFLs, AO is typically employed at the OGS to pre-distort the wavefront of the outgoing beam in order to pre-correct optical turbulence effects. However, to successfully perform AO mitigation, a proper measurement of turbulence-induced phase distortions along the same path traversed by the uplink beam must be performed. This is usually done by sensing the angle-of-arrival fluctuations of a received downlink beacon signal originating at the satellite. In this way, the conjugated phase to apply for pre-correction can be estimated^22^. The phase conjugation step is then performed through a fast-steering mirror that compensates for tilt errors and, in the case of higher-order AO, through a deformable mirror that corrects higher order distortions^26^. However, several limitations in the achievable correction exist.

First of all, the estimated correction is valid only over an angular distance equal to the isoplanatic angle. The latter quantifies the angular distance over which atmospheric turbulence remains unchanged, and depends on the turbulence profile, elevation angle, and wavenumber^17^. Nonetheless, consecutive downlink and uplink transmissions are angularly separated by the point-ahead angle (PAA). This is due to the finite speed of light and relative movement of the satellite with respect to the OGS. Thus, if the PAA is bigger than the isoplanatic angle, the AO system is degraded by anisoplanatism and the AO correction is not effective. This is particularly true for LEO OFLs, in which uplink and downlink paths can be completely uncorrelated^22^. However, anisoplanatism can also affect geostationary Earth orbit (GEO) OFLs because of the lateral speed between the OGS and the GEO satellite^27^.

In addition, the finite bandwidth of the AO system limits the maximum speed at which pre-distortion can be applied^26^. The rate at which AO corrections must be made is dictated by the Greenwood frequency, i.e., the rate at which atmospheric turbulence changes with time^17^. In the case of LEO OFLs, the spectrum of irradiance fluctuations comprises components at higher frequencies, up to several kHz, due to the satellite high speed^28^.

Finally, if the OGS transmits and receives through different apertures, the beam wander cannot be compensated and can be in the order of tens of microradians^22^.

In conclusion, beam wander plays a major role when transmitting an uplink untracked beam but can still lead to residual errors also in the case of tracked beams. Thus, its modeling in the proposed numerical tool is of major importance. In particular, regarding the scintillation index, both the model valid for an untracked beam and the one valid for a tracked beam, in which all beam wander effects have been perfectly removed, have been implemented. The related expressions can be found in^19^.

### Turbulence-induced beam spreading

Eddies smaller than the beam size lead to an additional diffraction of the propagating wave with respect to the one due to free space, as well as to a distortion of the received wavefront. In addition, the random movement of the short-term beam due to beam wander leads to a larger long-term spot size $W_{LT}$ at the receiver, whose square can be expressed as^17^

12 $$\begin{aligned} W_{LT}^2=W^2+W^2 T_{SS}+ W^2 T_{LS}. \end{aligned}$$

The first term in Eq. (12) is the beam radius at the receiver plane after propagation through free space. Together with the second term, that is associated with diffraction induced by small scale sizes, it defines the short-term beam radius $W_{ST}$.

Beam wander contributes to the long-term spot size through the third term in Eq. (12), that corresponds to the variance of the displacement of the instantaneous beam center at the receiver plane $\langle {r_c^2}\rangle$^17^.

The turbulence-induced beam spreading loss $L_{SR}$ can be evaluated through the Strehl ratio (SR), i.e., the ratio of the on-axis mean irradiance in presence of turbulence to the one in absence of turbulence at the receiver’s image plane^17^

13 $$\begin{aligned} L_{SR}=\left[ 1+\left( \frac{D_T}{r_0}\right) ^{\frac{5}{3}}\right] ^{-\frac{6}{5}}. \end{aligned}$$

It is important to highlight that this additional loss affects only the uplink propagation^17^.

### Pointing errors

The narrow beam divergence typical of optical beams and the limited field of view (FOV) of FSO receivers require a constant line of sight (LOS) connection between the transmitter and receiver optics. Thus, high pointing accuracies, in the order of sub microradians, must be ensured for the entire link duration to guarantee link availability^3^. However, several sources of pointing errors can affect OFLs, leading to deviations from the LOS between the OGS and the satellite and, thus, to a reduced received power, eventually causing link failure.

The narrow beam divergences and large distances involved in OFLs, combined with the relative motion between OGSs and satellites, make pointing a complex task. Therefore, automatic tracking systems must be properly designed and operated. Ephemeris data, i.e., the satellite position retrieved from the orbit equation, and navigation systems such as the global positioning system (GPS), are used to perform coarse pointing, while a tracking system enables fine pointing^29^.

Due to a Gaussian roll-off in the laser beam mean irradiance profile along the radial direction, even small pointing errors become significant in both propagation directions. Indeed, since scintillation levels increase with the square of the radial distance from the optical axis^17^, pointing errors can significantly impact fade statistics.

Pointing errors can be of three kinds, i.e., static, dynamic, and random errors. Static errors are fixed and do not depend on the link elevation angle. They consist of constant angular deviations $\Delta \theta _{OGS}$ (rad) from the LOS direction due to mechanical misalignments in the transmitter optics. Among sources of static errors, mechanical misalignments in the construction of the transmitter optics can be identified^30^.

Instead, dynamic errors are elevation dependent. Uncertainties in the LOS direction due to reference frame errors belong to this category^30^. Indeed, information extracted from GPS and two-line element (TLE) sets are not accurate enough to point an OGS towards a satellite. TLE files of satellite orbits can be downloaded from the U.S. Space Command’s Space-Track service^31^. However, the satellite could be distant from the predicted location due to uncertainty in these orbital elements, as well as due to time synchronization errors in the tracking system^32^. In detail, if the orbit information has been recently acquired, uncertainties are low. For instance, the mean along-track error (i.e., the error along the direction of the satellite trajectory), can vary from few meters when the TLE file is recent, up to more than 5 km after 3 days of satellite motion and more than 24 km after 7 days^30^.

Another elevation-dependent pointing error is the one due to an uncompensated PAA. The PAA value depends on the satellite orbital speed and elevation angle^30^. In a LEO OFL the PAA can range from about 20 $\upmu$rad at the horizon up to 50 $\upmu$rad at the zenith. Instead, the PAA for GEO OFLs varies between 17 $\upmu$rad and 20 $\upmu$rad, approximately^30^. Typically, a constant PAA correction is applied, leading to a residual pointing error. It can be demonstrated that the narrower the beam divergence and the higher the elevation, the higher the pointing loss^30^.

Random pointing errors, or jitter, consist of unpredictable errors that can be both elevation and non-elevation dependent, leading to uncertainties in the instantaneous direction of the beam with respect to the LOS. Thus, intensity fluctuations can be observed at the receiver.

Pointing jitter can be caused by noise in the tracking system and mechanical vibrations, other than by turbulence-induced beam wander. The satellite sources of vibrations can be internal or external^29^. The former encompass vibrations due to navigation noise, thruster operations, antenna pointing mechanism, solar array driver, noise in the tracking system and, generally speaking, operation of other satellite subsystems. The latter comprise the impact of micrometeorites, the gravitational fields of celestial bodies such as the Sun, the Moon, and the Earth, solar radiation pressure and satellite structure bending due to temperature gradients as well as to elastic forces of tension, and bending originating from the cyclic satellite movement^29^. The loss induced by deterministic pointing errors, i.e., PAA error, static errors, and orbit uncertainties, can be evaluated knowing the angular deviation related to them, as^30^

14 $$\begin{aligned} L_{pointing,static}=\exp {\left[ -2\left( \frac{\Delta \theta _{OGS}}{\frac{\theta _{e^{-2}}}{2}}\right) ^2\right] }. \end{aligned}$$

In Eq. (14), $\Delta \theta _{OGS}$ (rad) is the angular deviation induced by deterministic pointing errors and $\theta _{e^{-2}}/{2}$ (rad) is the radial angular distance from the beam center where the intensity falls to $1/e^2$ of its peak value, i.e., the half-divergence angle. The latter can be derived from the transmitter beam radius as^16^

15 $$\begin{aligned} \frac{\theta _{e^{-2}}}{2}=\frac{\lambda }{\pi W_0} \end{aligned}$$

and is also related to the full width at half maximum (FWHM) divergence angle by the following equation^16^

16 $$\begin{aligned} \frac{\theta _{e^{-2}}}{2}=\frac{\theta _{FWHM}}{\sqrt{2\ln {2}}}. \end{aligned}$$

In presence of random errors, the radial pointing error can be statistically described by a Rician distribution if azimuth and elevation errors are identically distributed and uncorrelated^33^. If the bias error is zero, the distribution simplifies to a Rayleigh one. In the last case, the jitter-induced pointing loss is expressed as^30^

17 $$\begin{aligned} L_{pointing,random}=\frac{\left( \frac{\theta _{e^{-2}}}{2}\right) ^2}{\left( \frac{\theta _{e^{-2}}}{2}\right) ^2+4 \sigma _{jitt}^2} \end{aligned}$$

where $\sigma _{jitt}$ can account for both vibrations and beam wander. Indeed, assuming that these random errors are independent and normally distributed, their standard deviations can be added in quadrature to evaluate the total pointing jitter^30^.

Nonetheless, the simultaneous occurrence of constant bias errors and random errors can have a huge impact on BER performance^33^. Thus, also a comprehensive model for pointing loss accounting for both constant and random errors has been implemented in the tool. According to^30^, the average pointing loss becomes

18 $$\begin{aligned} L_{pointing,static+random}=\frac{\left( \frac{\theta _{e^{-2}}}{2}\right) ^2}{\left( \frac{\theta _{e^{-2}}}{2}\right) ^2+4 \sigma _{jitt}^2} \cdot \exp \left[ -2\frac{\Delta {\theta ^2_{tot}}}{{\left( \frac{\theta _{e^{-2}}}{2}\right) ^2+4 \sigma _{jitt}^2}}\right] . \end{aligned}$$

The developed numerical tool gives the possibility to choose if considering both static and random pointing errors or only one of them.

The flexible modeling of pointing errors is of great importance to design OFLs. Indeed, sufficient control bandwidth and dynamic range must be allocated to deal with pointing errors and jitter. Moreover, the accuracy of the pointing and tracking system must be comparable with the beam divergence angle. The latter could be increased to counteract pointing uncertainties. However, this strategy could lead to unacceptable low received power and, thus, to a negative link margin.

### Fog and clouds

Adverse weather conditions associated with the presence of hydrometeors can affect the laser beam propagation. Unlike RF signals, which are mostly degraded by the rain, the major threat for optical beams is represented by fog due to the comparable size of the droplets with the optical wavelengths, leading to a high scattering efficiency. The Mie theory can be adopted to derive the related attenuation, that can reach values up to 480 dB/km^34^, making the link temporarily unfeasible.

Since the presence of clouds affects the propagation in a similar way, the combined phenomena can be modelled through an extinction coefficient $\beta _{fog/clouds} (\lambda )$ (km$^{-1}$)

19 $$\begin{aligned} \beta _{fog/clouds}(\lambda )=\frac{3.91}{V}\left( \frac{\lambda }{0.55}\right) ^{-q} \end{aligned}$$

where $V$ (km) is the visibility and the wavelength is expressed in micron. Several empirical models are available for the $q$ exponent, that is the so-called size distribution coefficient of scattering. Our tool implements some of the most validated models, that are the Kruse model, the Kim one, and the Al Naboulsi one^34^. The first one’s validity is not sure for visibility values less than 1 km. Therefore, the second one is a modified version of the first one able to overcome this limit^34^. Finally, the Al Naboulsi model is related only to fog attenuation and is valid for wavelengths between 0.69 $\upmu$m and 1.55 $\upmu$m and for visibilities between 50 m and 1 km. It directly allows to compute a specific attenuation $A_{fog}$ (dB/km), from which the overall fog-related loss $L_{fog}$ (dB) can be retrieved as

20 $$\begin{aligned} L_{fog}=A_{fog} l_{fog}. \end{aligned}$$

Instead, starting from $\beta _{fog/clouds}(\lambda )$ the related loss $L_{fog/clouds}$ (dB) is given by

21 $$\begin{aligned} L_{fog/clouds}=10\log \,_{10}\exp (-\beta _{fog/clouds}(\lambda ) l_{fog/clouds}). \end{aligned}$$

In Eqs. (20) and (21), $l_{fog}$ and $l_{fog/clouds}$ represent the in-fog/clouds propagation lengths expressed in kilometers.

### Rain

Raindrops size results larger than the signal wavelength in the optical domain, leading to a wavelength-independent scattering of the optical wave traversing them. The related loss is of the same order of the one affecting millimeter-waves, ranging from about 1–10 dB/km^3^. Therefore, models developed for millimeter-waves can be adopted also in the optical domain.

The rain extinction coefficient $\beta _{rain}$ (dB/km) is a function of the rain rate $R$ (mm/h) through the following relationship^3^

22 $$\begin{aligned} \beta _{rain}=\gamma ^{\alpha } \end{aligned}$$

in which $\gamma$ and $\alpha$ are parameters dependent on the raindrop-size distribution whose values have been experimentally established in different models. The proposed numerical tool implements the Marshall and Palmer^35^, the Carbonneau^9^, and the Korai, Luini and Nebuloni (KLN) models^36^. As an alternative, also a model based only on visibility, i.e., the Atlas one^37^, has been implemented. As for the fog loss, the rain loss $L_{rain}$ (dB) is obtained multiplying Eq. (22) for the in-rain propagation length $l_{rain}$ (km).

### Snow

Usually, the size of the snowflakes is bigger than that of the raindrops, resulting in higher attenuation of the optical wave, ranging from 3 to 30 dB/km^16^, with also larger values if the snow completely hinders the optical beam. The snow specific attenuation $\beta _{snow}$ (dB/km) can be evaluated as^9^

23 $$\begin{aligned} \beta _{snow}=a S^{b} \end{aligned}$$

where $S$ (mm/h) is the snowfall rate and $a$ and $b$ parameters, whose expressions differ between wet and dry snow, depend on the wavelength^9^. Alternatively, an empirical model based only on visibility can be adopted^38^. Also in this case, an in-snow propagation length $l_{snow}$ (km) is needed to compute the overall snow loss $L_{snow}$ (dB).

### Transmitter and receiver

The transmitter impacts on the link budget through the laser source emitted optical power $P_T$ (W), the transmitter chain efficiency $\eta _T$ and the telescope gain $G_T$. This last term is strictly related to the wavelength and the telescope aperture diameter and can be derived starting from the solid emission angle $\Omega _T$ (srad)^38^, leading to

24 $$\begin{aligned} G_T=\frac{4\pi }{\Omega _T}\cong \left( \frac{4 D_T}{\lambda }\right) ^2. \end{aligned}$$

The receiver telescope also amplifies the signal through its gain $G_R$, which is still dependent on the telescope aperture diameter $D_R$^38^

25 $$\begin{aligned} G_R=\left( \frac{\pi D_R}{\lambda }\right) ^2. \end{aligned}$$

For the receiver, as for the transmitter, an efficiency $\eta _R$ must be taken into account. Finally, if the received beam needs to be coupled in a single mode fiber (SMF) prior to its conversion in the electrical domain for being amplified, the link budget equation will include a SMF coupling efficiency $\eta _F$, that is not considered for non-pre-amplified intensity modulation/direct detection schemes.

Optical turbulence downgrades the spatial coherence of the waves, limiting the achievable SMF coupling efficiency in reception. A model for this term is the one given in^39^, that is

26 $$\begin{aligned} \eta _F= 8a^2 \int _{0}^{1} \int _{0}^{1} \exp \left[ \left( -a^2-\frac{A_R}{A_C}\right) \left( x_1^2+x_2^2\right) \right] \cdot I_0\left( \frac{2A_R}{A_C} x_1 x_2\right) x_1 x_2 \,dx_1\,dx_2. \end{aligned}$$

In Eq. (26), $A_R$ (m$^{2}$) is the receiver aperture area and $A_c=\pi \rho _0^2$ (m$^{2}$) is the spatial coherence area of the incident wave, also-called speckle size, being $\rho _0=0.48 r_0$ (m) the spatial coherence radius^17^, and $I_0(x)$ is the modified Bessel function of the first kind and zero order. Instead, the $a$ parameter depends on the receiver specifications as follow:

27 $$\begin{aligned} a=\frac{D_R}{2} \frac{\pi W_m}{\lambda f} \end{aligned}$$

where $W_m$ (m) is the fiber-mode field radius at the fiber end face and $f$ (m) is the receiver focal length.

Equation (26) is valid under the approximation of a Gaussian field mutual coherence function but its accuracy, evaluated in^17^, is such that it is worth to implement this model with respect to the most general one, allowing also to reach a trade-off between accuracy and computational cost.

## Link budget analysis for experimental OFLs

In this section, the numerical results related to the link budget analysis for two different OFLs scenarios, one considering a downlink transmission from a LEO satellite and the other analyzing the uplink communication towards a GEO satellite, are shown.

The models presented in the previous section have been integrated to build a novel link budget model, which is able to properly evaluate their combined impact in each turbulence regime. In particular, the great variety of implemented models allows to adequately evaluate the impact of phenomena which are overlooked in existing link budget equations, which are not meant to quantify the combined impact of scintillation and beam wander^8–10,14^, as well as of random and deterministic pointing errors^8–14^, which are interrelated phenomena.

In addition, the possibility to choose among different perturbed sky effects-related empirical models potentially extends the tool applicability to any geographic location.

The proposed command-line numerical tool takes as input some system parameters fully characterizing the scenario of interest as well as the models to be used in the computation and returns in output all the gain and loss terms together with the mean received optical power and the estimated necessary fade margin.

Given the computed mean received optical power $P_R$ (dB), if $S_R$ (dB) is the receiver sensitivity, the available link margin $LM$ (dB) can be evaluated as

28 $$\begin{aligned} LM=P_R-S_R \end{aligned}$$

where $P_R$ (W), in the most general case, is evaluated by the tool as

29 $$\begin{aligned} P_R=P_TG_T\eta _T\eta _R\tau _{ATM}L_{pointing}L_{SR}L_{FS} \cdot L_{fog/clouds}L_{rain}L_{snow}. \end{aligned}$$

While in uplink $L_{pointing}$ accounts for both beam wander and other kinds of pointing errors, in downlink beam wander is neglected. In the same way, the turbulence-induced beam spreading loss $L_{SR}$ must not be considered in downlink.

The link margin must be able to cope with fading and is related to the link availability. Indeed, the higher the link margin, the higher the link availability. However, the adopted modulation, coding, and eventual diversity scheme define the receiver sensitivity^3^. Thus, the link availability that a certain link margin can guarantee depends on the particular system configuration.

The tool gives also the possibility to perform parametric sweep analysis defining an array of values for up to two input parameters, which can be helpful to understand how the OFL design changes in different operating conditions and to optimize the system. In this case, the output quantities are returned specifying to which values of the variable input parameters they correspond to. The sweep capabilities are also shown in this section through the link budget analysis of a dynamic downlink transmission from a LEO satellite. Indeed, due to the relative movement of the LEO satellite with respect to the OGS, the link geometry changes over time leading to a variable received optical power.

### Downlink LEO OFL

The Japan Aerospace Exploration Agency (JAXA), together with the NICT, conducted the first bi-directional in-orbit OFL demonstration using a LEO satellite, in 2006^40^.

The Kirari Optical Communication Demonstration Experiments with the NICT OGS (KODEN) took place during March, May and September 2006, as well as during October 2008 and February 2009^41^. In details, the transmission between the Optical Inter-orbit Communications Engineering Test Satellite (OICETS) and the NICT OGS in Koganei, Tokyo, was performed considering one beam for the downlink transmission and a multi-beam system, based on four laser beams in order to mitigate the scintillation effect, for the uplink transmission. In this paper, the downlink transmission was numerically validated.

The OICETS, orbiting at an altitude $H=610$ km, is equipped with the Laser Utilizing Communications Equipment (LUCE) featuring a telescope with a transmitting aperture diameter $D_T=26$ cm and transmitting at a wavelength $\lambda =847$ nm with a power $P_T=100$ mW and an efficiency $\eta _T = -2.7$ dB.

The NICT OGS, located at an altitude $h_{0GS}=122$ m, featured an optical receiver employing a receiving telescope with an aperture diameter $D_R=20$ cm and presenting an efficiency $\eta _R=-8.1$ dB. A sub-aperture of the 1.5 m transmitting telescope, with diameter $D_R=31.8$ cm, was used for reception after May 2006^41^. Since one goal of KODEN was to evaluate the degradation of the SMF coupling efficiency in downlink transmissions^42^, this loss term must be considered in the link budget analysis.

Results from measurements taken on 30 March 2006, are reported in^40^. Since there are no clear indications on the acquisition timing, which would allow to reconstruct the plot of the downlink received optical power for the entire link duration starting from the knowledge of the satellite orbit, the link budget has been evaluated only for a particular system configuration, i.e., considering an elevation angle of 25$^\circ$, for which measurements of the atmospheric transmittance are available^40^.

Given that the considered field test happened before May 2006, the link budget analysis reported here adopts the 20 cm receiving aperture diameter as one of the input parameters.

Regarding the input parameters characterizing the channel, a transmittance equal to 0.45, consistent with the value in^40^, has been considered. For what concerns the turbulence strength, a $C_n^2 (h)$ model was developed for the NICT OGS based on measured data^41^. Its expression is a modification of the HV model, given by^41^

30 $$\begin{aligned} C_n^2(h)=M\cdot 0.00594\left( \frac{w}{27}\right) ^2 (10^{-5}h)^{10} \exp \left[ -\frac{h}{1000}\right] +2.7\cdot 10^{-16}\exp \left[ -\frac{h}{1500}\right] +A\exp \left[ -\frac{h}{100}\right] \end{aligned}$$

where the parameters were estimated to be $M=0.2$ and $A=9.0\times 10^{-14}$ m$^{-2/3}$, with $w=21$ m/s. Thus, the tool has been extended to also consider this model for the refractive index structure parameter. In this way, a comparison between the measured quantities and the computed ones can be performed.

Given the not so high elevation angle, which can lead to a major impact of turbulence on the beam propagation, the extended Rytov theory has been chosen to compute the turbulence-related quantities. In particular, for the scintillation margin the rigorous model has been used giving in input to the tool a value of the desired fraction of outage time $\rho _{thr}=10^{-4}$, which corresponds to a link availability of 99.99%.

No perturbed sky effects have been included since the measured transmittance value already accounts for the partly cloudy weather^40^.

During each experiment, the initial bias pointing error was eliminated by entering offset commands at the OGS. Only after achieving alignment, the communication phase could start.

Measurements of the fine tracking error made on 30 March 2006 are reported in^41^, according to which the tracking error was calculated to be $\sigma _{jitt}=2.0$ $\upmu$rad.

The link budget analysis for the downlink LEO OFL is shown in Table 1.

**Table 1:** Link budget analysis for downlink LEO OFL.

Output parameter	Downlink LEO OFL
Transmitted power $P_T$	20 dBm
Free space loss $L_{FS}$	− 265.32 dB
Transmitter telescope gain $G_T$	121.78 dB
Receiver telescope gain $G_R$	117.41 dB
Extinction loss $\tau _{ATM}$	− 3.80 dB
Turbulence-induced beam spreading loss $L_{SR}$	–
Pointing loss $L_{pointing}$	− 2.53 dB
Scintillation margin $L_{SI}$	− 9.13 dB
SMF coupling efficiency $\eta _F$	− 13.74 dB
Received optical power $P_R$	− 36.98 dBm

The computed mean received optical power, equal to − 36.98 dBm, agrees with the experimentally measured values depicted in^40^. Moreover, the value of the computed SMF coupling efficiency, i.e., − 13.74 dB, falls in the range of the measured values that goes from − 11 to − 18 dB, as shown in^42^.

Regarding turbulence, a Fried’s parameter $r_0=4.62$ cm and a scintillation index $\sigma ^2_I=0.32$ can be retrieved from the numerical tool which, for the computation of the latter, has considered aperture averaging at the receiver. Indeed, the tool has verified that the estimated transverse correlation width results smaller than the receiver aperture diameter. The two computed values agree with the measured ones plotted in^41^.

From the computed scintillation margin, it is evident that scintillation must be carefully taken into account during system design activities since it can introduce a significant performance degradation. Indeed, it is necessary to ensure that the received power stays above the receiver sensitivity to make the link feasible which, in the case of a LEO satellite, implies that this condition must be satisfied for different elevation angles due to the satellite movement during the entire link duration.

### Uplink GEO OFL

Regarding the uplink transmission direction, the communication between the Tesat’s Transportable Adaptive Optical Ground Station (T-AOGS) and the TDP1 laser communication terminal (LCT) onboard Alphasat satellite has been analyzed^43^.

The same scenario of the experimental measurements made on 26 April 2018, is considered for the link budget estimation. Starting from the TLE relative to the Alphasat orbit, downloadable at^31^, and from the knowledge of the OGS coordinates, which is co-located with the ESA OGS in Tenerife at an altitude $h_{OGS}=2450$ m^43^, the link distance, the satellite altitude, and the elevation angle can be retrieved in Matlab, by means of built-in functions, for a given time instant. Specifically, the start time of the acquisition has been chosen for the analysis, corresponding to 01:05:48 UTC, leading to a satellite altitude $H=35{,}800$ km, an elevation angle of 32.9$^\circ$, and a link distance $L=38{,}368$ km.

The T-AOGS can transmit an optical beam through three different aperture diameters, i.e., 35 mm, 48 mm, or 95 mm^43^, with optical power $P_T=50$ W and efficiency $\eta _T=-1.5$ dB at the wavelength $\lambda =1064$ nm^44^. In the mentioned experiment, the smaller aperture has been used, which implies a transmitter beam radius $W_0=15.6$ mm^44^. Instead, the TDP1-LCT is equipped with a receiving telescope with an aperture diameter of 135 mm and adopts coherent homodyne detection^43^, thus SMF coupling efficiency is considered in the link budget.

For what concerns pointing errors, the tracking system of the LCT shows a residual tracking error during communications with root mean square value $\sigma _{jitt}=0.07$ $\upmu$rad^45^. At the same time, the T-AOGS alignment was adjusted for each link by an operator, reducing the static pointing error up to $\Delta \theta _{OGS}=10$ $\upmu$rad^46^.

Regarding the atmospheric channel, a transmittance equal to 0.92, extrapolated from other measurements done in the same location in similar conditions^47^, has been considered. In addition, no perturbed sky effects have been included in the link budget estimation.

Regarding the turbulence model, the Maui3 profile, developed for another OGS site with characteristics similar to the Tenerife one, results to be the most representative of measurement data^46^. Thus, as for the previous scenario, the tool has been extended implementing this model, whose mathematical expression can be found in^48^. In this way, a fair comparison between numerically evaluated and measured quantities can be performed.

The extended Rytov theory has been adopted to compute turbulence-related quantities. This last choice is due to the same observation made for the downlink LEO OFL related to the not so high elevation angle.

The scintillation margin is evaluated through the rigorous expression considering a 99.99% link availability and an untracked beam.

The link budget analysis, reported in Table 2, returns a mean received optical power of − 43.94 dBm, very close to the measured mean value of − 42.6 dBm^43^.

**Table 2:** Link budget analysis for uplink GEO OFL.

Output parameter	Uplink GEO OFL
Transmitted power $P_T$	46.99 dBm
Free space loss $L_{FS}$	− 293.13 dB
Transmitter telescope gain $G_T$	102.38 dB
Receiver telescope gain $G_R$	112.01 dB
Extinction loss $\tau _{ATM}$	− 0.36 dB
Turbulence-induced beam spreading loss $L_{SR}$	− 0.68 dB
Beam wander and pointing loss $L_{pointing}$	− 5.16 dB
Scintillation margin $L_{SI}$	− 9.74 dB
SMF coupling efficiency $\eta _F$	− 4.50 dB
Received optical power $P_R$	− 43.94 dBm

The tool also returns a Fried’s parameter $r_0=11.46$ cm and a scintillation index $\sigma _I^2=0.37$. These values are in agreement with the measured ones shown in^43^, validating the tool accuracy.

### Dynamic downlink LEO OFL

The German Aerospace Center (DLR) cooperated with JAXA and NICT to perform the first LEO downlink transmission in Europe during 2006, in the framework of the KIrari’s Optical Downlink to Oberpfaffenhofen (KIODO) experiment. In particular, the LUCE terminal onboard OICETS was exploited to transmit an optical beam towards a ground station located in Oberpfaffenhofen (OGS-OP), near Munich^49^.

**Figure 3:**
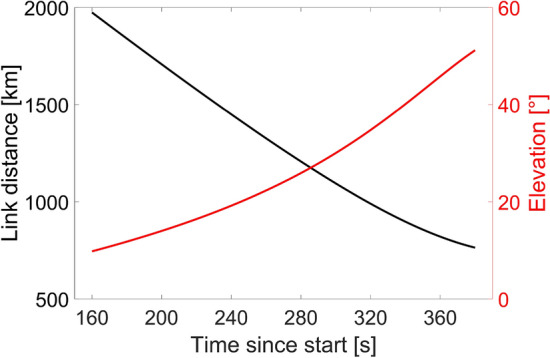
Link distance (black curve) and elevation angle (red curve) as a function of time elapsed since the pass of the satellite for the dynamic downlink LEO scenario.

The OFL involved in the experiment, which took place on 14 June 2006, has been analyzed through the developed tool. Due to the knowledge of the exact acquisition start time, i.e., 01:04 UTC, and experiment runtime, i.e., 380 s, which can be found in^49^, and thanks to sweep capabilities of the tool, the received optical power at the OGS-OP has been evaluated for the entire link duration. As for the uplink GEO OFL scenario, the satellite orbit reconstructed in Matlab starting from the related TLE file^31^, and the coordinates of the OGS-OP^50^, have been used to compute the link distances and elevation angles for the entire link duration considering a 1 Hz sampling frequency. Figure 3 shows the link distance (black curve) and elevation angle (red curve) as a function of the time elapsed from the start of the pass of the satellite. As expected, due to the dynamic movement of the LEO satellite, the link distance and elevation angle change over time leading to higher link distances for lower elevation angles.

The link budget analysis has been evaluated considering the transmission of a 100 mW beam at a wavelength of 847 nm from a telescope with aperture diameter $D_T=26$ cm as for the downlink transmission between OICETS and the OGS located in Koganei^40^. Instead, the OGS-OP is equipped with a receiving telescope characterized by $D_R=40$ cm^49^, followed by a direct detection chain which implies that the SMF coupling efficiency must not be included in the link budget^50^. Since no measurements of the transmitter and receiver efficiencies for this experiment are available in literature, a transmitter efficiency of − 2.7 dB, equal to the one involved in the previously analyzed downlink transmission from the same optical terminal, has been considered^40^, while $\eta _R$ is set equal to 0 dB.

For the considered experiment, the maximum root mean square tracking error is known to be $\sigma _{jitt}=0.7$ $\upmu$rad^51^. Regarding the atmospheric transmittance, a value of 0.19 has been considered as in^51^.

Measurements of the refractive index structure parameter for each trial of the KIODO experiment are available in^51^. Moreover, the best fit turbulence profile was estimated based on the scintillation indexes measured during 2006 trials^52^. The outcomes of the fitting procedure show that the HV model with $A=2.20\times 10^{-12}$ m$^{-2/3}$ and $w=10$ m/s and the extended Rytov theory return the better results.

Since the measurements took place during a clear sky night^49^, no perturbed sky effects have been modelled.

**Figure 4:**
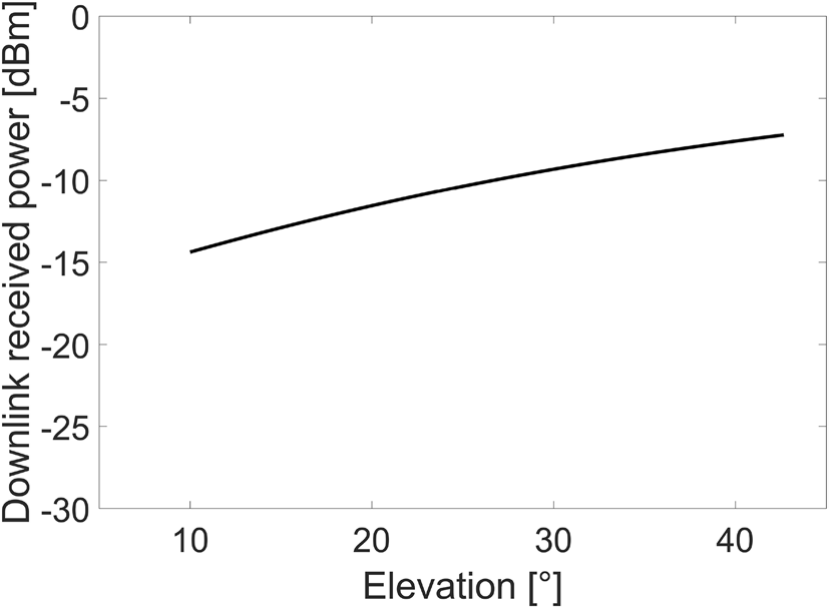
Received optical power as a function of the elevation angle for the dynamic downlink LEO scenario.

Figure 4 depicts the downlink computed received optical power as a function of the elevation angles involved in the communication timeframe.

The computed received optical power exhibits the same behavior of the measured one reported in^49^, except for the fact that the obtained values are higher than the measured ones of about 8 dB. However, the approximations made for the receiver and transmitter efficiencies, due to the lack of measurement data, can explain this discrepancy.

**Figure 5:**
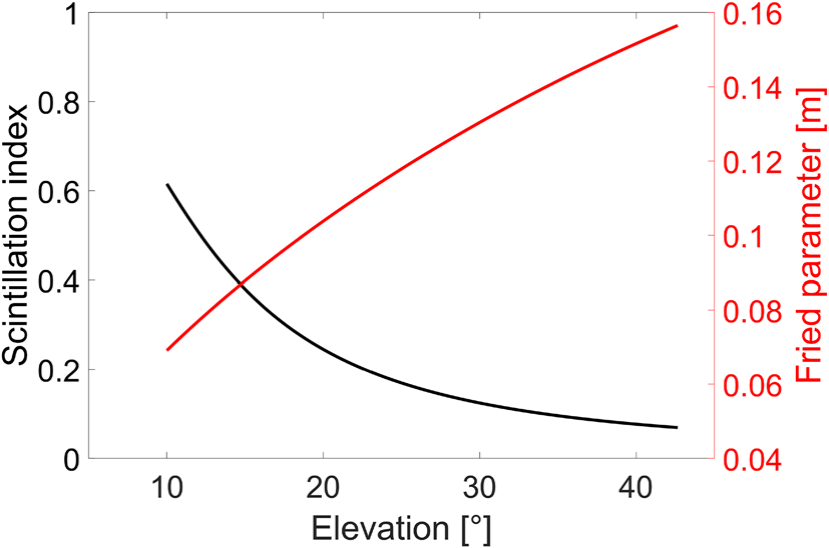
Scintillation index (black curve) and Fried parameter (red curve) as a function of the elevation angle for the dynamic downlink LEO scenario.

From the computed received optical power it is evident that a LEO OFL with no adjustments of the transmitted power, according to the variable link distance, is characterized by a variable received power. Therefore, the link must be properly designed to guarantee a received power higher than the receiver sensitivity for the entire link duration, especially for lower elevation angles or, in the same way, the transmitted power must be properly varied to obtain a constant received signal. To further validate the accuracy of the performed link budget analysis, the Fried’s parameter and scintillation index have been extracted and plotted in Fig. 5 in red and black curves, respectively, as a function of the elevation angle.

From the black curve of Fig. 5, it is possible to note that a higher elevation angle leads to a reduced scintillation since the portion of the beam propagation path traversing the atmosphere becomes smaller.

Finally, the Fried’s parameter, plotted in the red curve of Fig. 5 as a function of the elevation angle, further confirms that a higher elevation angle is associated with a reduced turbulence effect on the beam propagation leading to a larger atmospheric coherence length.

Both the scintillation index and the Fried’s parameter show behaviors in agreement with the measured ones^49^, validating the tool capability to accurately predict the link performances. However, numerical values perfectly correspond to measured ones only for the scintillation index^49,51^, while a slight overestimation of the Fried’s parameter is obtained from the tool. This can be explained considering that the adopted turbulence profile has been optimized only based on measured scintillation indexes and not considering the measured Fried’s parameters^52^.

## Impact of link distance on the beam wander for experimental OFLs

In the uplink GEO OFL analyzed in the previous section, the beam wander loss has been evaluated through the conventional model, i.e., the implementation of Eq. (7) with the conventional approximation of Eq. (10) for the link distance has been used.

A comparison between the results previously presented and the ones obtained substituting the exact link distance and the more accurate approximation of Eq. (11) has been carried out for the same GEO scenario, i.e., assuming the same inputs. In particular, the same geometrical and channel parameters enumerated in the “Uplink GEO OFL” section have been adopted. Table 3 shows the outcome of this analysis. In particular, as explained above, the exact link distance value has been evaluated through built-in Matlab functions which require in input the satellite TLE file, necessary to reconstruct its orbit, and the OGS coordinates.

**Table 3:** Beam wander parameters for uplink GEO OFL.

Link distance	Output parameter		
	Beam wander variance $\langle {r_c^2}\rangle$	Angular beam wander $\theta _{BW}$	Beam wander loss $L_{BW}$
Conventional approximation ${L}=65,908.14$ km	$3.1203\times 10^5$	14.5590 $\upmu$ rad	− 4.4787 dB
More accurate approximation $L=38,368.91$ km	$1.0575\times 10^5$	8.4757 $\upmu$ rad	− 2.0726 dB
Exact value $L=38,367.80$ km	$1.0574\times 10^5$	8.4757 $\upmu$ rad	− 2.0726 dB

The obtained results suggest that the conventional model leads to an overestimation of the beam wander variance and angular beam wander, leading to a worse beam wander loss with respect to the one evaluated adopting the exact link distance value for both the numerator and denominator of Eq. (9).

Instead, the more accurate approximation allows to correctly evaluate the three beam wander parameters since it returns a link distance which differs from the exact value of only about 1 km, that corresponds to an error of 0.0029% against a 71.78% error related to the conventional link distance approximation. Thus, preliminary link design procedures, characterized by not known OGS coordinates and satellite orbit parameters, can benefit from the high achieved accuracy resulting in enhanced link budget analyses.

Considering the absolute error between the two approximations shown in “Beam wander” section, for elevation angles smaller than the one involved in the chosen uplink GEO scenario, a bigger error in the evaluation of the beam wander parameters is expected. This problem can mostly affect uplink communications with LEO satellites, since the elevation angle changes during the link duration and can reach low values.

Therefore, the beam wander has been analyzed also for the uplink communication in the same scenario of the downlink LEO OFL presented in “Downlink LEO OFL” section, but considering the transmission of a single beam with respect to the real measurements. In details, the NICT OGS transmits at a wavelength $\lambda =815$ nm with a transmitter beam radius $W_0=0.25$ cm^40^.

Regarding the acquisition timeframe, necessary to compute the OICETS orbit, the link distance and the elevation angle, the 30 March 2006 trial has been considered as for the downlink budget analysis. It is known that the link started around midnight (local time) and lasted 4 min and 41 s, but the exact acquisition start time is not specified in literature^40^. Given these considerations and the fact that the transmission was allowed only for elevation angles above 15$^\circ$ for safety reason^41^, a start time equal to 00:36:15 (local time) was chosen. Doing so, elevation angles between 16.02$^\circ$ and 47.67$^\circ$ are involved in the simulated scenario. The channel has been modeled considering the same parameters adopted for the “Dynamic downlink LEO OFL” scenario.

**Figure 6:**
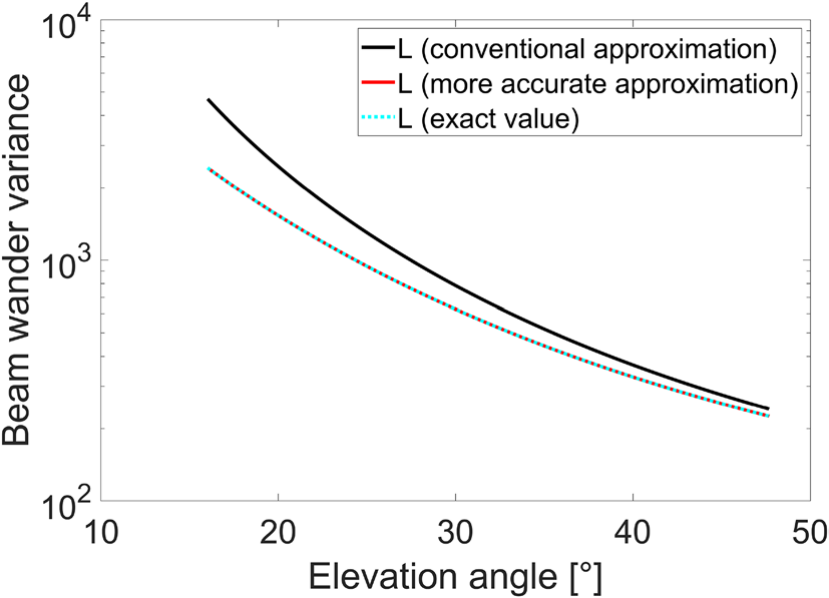
Beam wander variance as a function of the elevation angle for uplink LEO scenario, evaluated considering the conventional link distance approximation (black curve), the more accurate link distance approximation (red curve) and the exact link distance value (dashed cyan curve).

**Figure 7:**
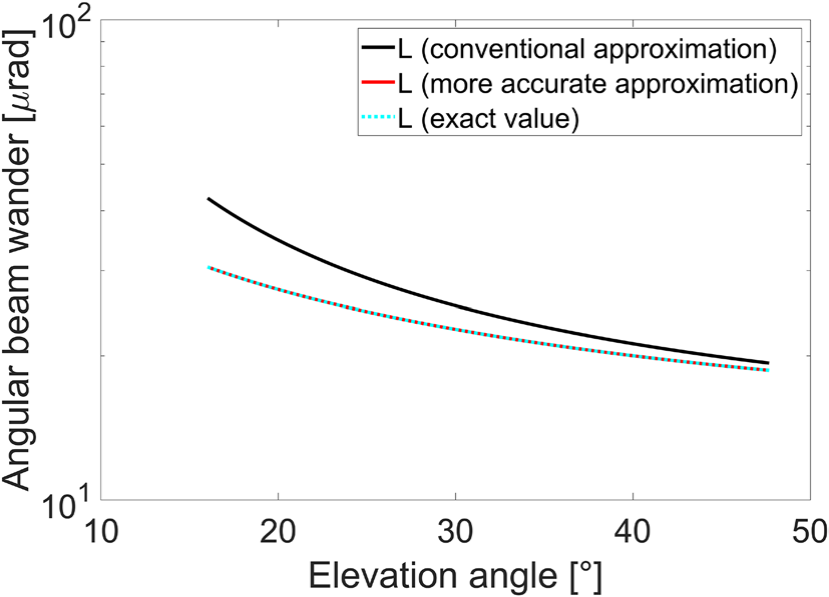
Angular beam wander as a function of the elevation angle for uplink LEO scenario, evaluated considering the conventional link distance approximation (black curve), the more accurate link distance approximation (red curve) and the exact link distance value (dashed cyan curve).

**Figure 8:**
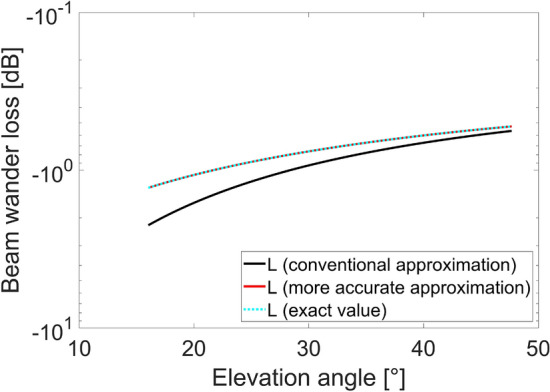
Beam wander loss as a function of the elevation angle for uplink LEO scenario, evaluated considering the conventional link distance approximation (black curve), the more accurate link distance approximation (red curve) and the exact link distance value (dashed cyan curve).

The beam wander variance, angular beam wander and beam wander loss, for the proposed uplink LEO scenario, are reported in Figs. 6, 7, and 8, respectively. Specifically, the black curves consider the conventional approximation for the link distance, the red ones are obtained substituting the more accurate link distance approximation in Eq. (7) and the cyan curves derive from the use of the exact link distance value. For all three figures, the last two curves are overlapped, confirming the accuracy of the more accurate approximation.

As expected, the overestimation behavior of the conventional beam wander model is higher for lower elevation angles while it reduces as the elevation angle increases, reflecting the trend observed in “Beam wander” section for the error between the two link distance approximations.

The wrong evaluation of the beam wander effect can lead to an overestimation of the resulting loss at the receiver and, above all, to a wrong prediction of the hot spot displacement which needs to be corrected to guarantee a good alignment between the transmitter and the receiver during the communication.

## Conclusions

In this paper, a novel numerical tool for link budget estimation has been presented in response to the need to have an instrument able to support in a flexible, accurate and efficient way the design of OFLs, which are expected to be a key element in future telecommunication networks. This task requires to fully understand the electromagnetic phenomena affecting the optical beam propagation from ground to space and from space to ground as well as the interrelationships between them. A comprehensive survey has been reported alongside the related main mathematical models implemented in the proposed tool, leading to a novel and comprehensive link budget model.

The tool capabilities have been exhaustively presented and validated through the analysis of three experimental OFLs for which measurements results are available in literature. To compare computed quantities with experimental ones, turbulence conditions resembling the ones experienced during measurements have been considered. Nonetheless, during the procedure of designing an OFL, link budget evaluations should be carried out considering a worst-case scenario. This can be done by means of the developed tool, choosing the proper model for the refractive index structure parameter among the implemented ones.

Moreover, a novel analysis of the conventional beam wander model and its limitations has been performed, thanks to the tool capability to compute both the exact link distance value and a more accurate approximation other than the conventional coarse approximation. In particular, the overestimation behavior of the beam wander model, leading to incorrect link budget analyses, has been demonstrated and overcome. Consequently, the tool properly allows to support system design activities with an enhanced accuracy, especially for low elevation angles.

The proposed tool could be adopted and extended to analyze FSO links with other platforms, such as HAPs, UAVs or ships, which can be helpful in the context of 3-D networks.

## Data Availability

Data underlying the results presented in this paper are not publicly available at this time but may be obtained from the authors upon reasonable request.
